# Organizing pneumonia as an initial presentation in a male lupus: A rare case report

**DOI:** 10.1002/ccr3.7389

**Published:** 2023-05-18

**Authors:** Suman Acharya, Roshan Aryal, Rupesh Kumar Yadav, Suraj Shrestha, Rikesh Karki, Saket Jha

**Affiliations:** ^1^ Department of Internal Medicine Tribhuvan University Teaching Hospital, Institute of Medicine Kathmandu Nepal; ^2^ Maharajgunj Medical Campus Institute of Medicine Kathmandu Nepal; ^3^ Nepalese Army Institute of Health Science Kathmandu Nepal; ^4^ Kathmandu Medical College and Teaching Hospital Kathmandu Nepal

**Keywords:** interstitial lung disease, lupus, organizing pneumonia

## Abstract

Organizing pneumonia (OP) is one of the rare pulmonary manifestations of systemic lupus erythematosus (SLE) which is infrequently reported as a presenting manifestation. Early diagnosis of lupus‐related OP with the help of imaging, can drive to prompt initiation of immunosuppressive therapy leading to a better prognosis. We present a case of a 34‐year‐old young male who presented with fever, myalgia, and a dry cough for 1 month and was later diagnosed as SLE‐related organizing pneumonia.

## 1 INTRODUCTION

Pulmonary involvement is frequent in patients with systemic lupus erythematosus (SLE) with the occurrence ranging from 24% to 74%, but the patients presenting with the pulmonary implication as the initial manifestation of SLE account for only 2%–4% of the cases.^1^ Organizing pneumonia (OP), a form of interstitial lung disease (ILD) characterized by fibrosis and scarring of the lung, as a presenting feature in SLE that too in male patients is infrequent and has been rarely narrated in literature.^1^, ^2^, ^3^

Here we report a case of a 34‐year‐old man who presented with OP as an initial manifestation of SLE.

## 2 CASE REPORT

**FIGURE 1:**
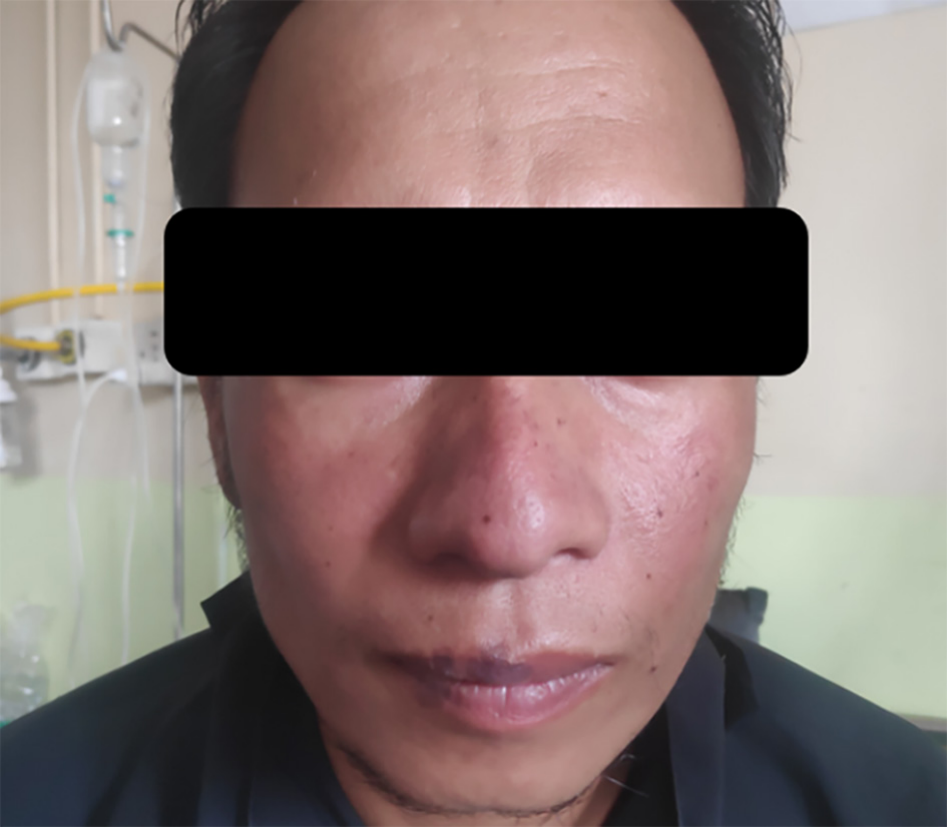
Photosensitive facial rashes over malar prominences and bridge of nose.

A 34‐year‐old young man with a history of recently diagnosed diabetes mellitus presented to our center with complaints of fever and generalized muscle ache for 1 month. He also had a dry cough, pleuritic‐type chest pain for the same duration, and clinically significant weight loss. Photosensitive facial rashes were present as shown in Figure 1. There were no other symptoms of lupus such as joint pain, hair loss, oral ulcer, etc.

On admission, he was febrile (temperature: 100°F), tachypneic (respiratory rate: 30 breaths/min), tachycardic (pulse rate: 108 beats per minute) with low oxygen saturation (SpO2: 87% in room air) but with normal blood pressure. Auscultation revealed fine crackles in the bilateral basal lungs. The rest of the examinations were unremarkable.

Laboratory investigations showed hemoglobin of 11.6 g/dL, a total count of 6700/mm^3^, and a platelet count of 81,000/mm^3^ with normal liver and kidney functions. Urinalysis revealed proteinuria (2.2 g/24 h) and glycosuria. He had a C‐reactive protein (CRP) level of 12.8 mg/L and an erythrocyte sediment rate (ESR) of 52 mm/h. Serum biochemistry profile showed HbA1c of 11.6% without other abnormalities.

Serological antibody testing for common respiratory pathogens, and blood culture did not reveal any abnormalities. Immunological testing was positive for antinuclear antibody (ANA) (197.21 IU/mL), anti–double‐stranded deoxyribonucleic acid (anti‐dsDNA) antibody (168.1 IU/mL), anti‐smith (anti‐Sm) antibody (95 RU/mL) with low complement levels of C3 (12.7 mg/dL, normal range: 90–180 mg/dL) and C4 (8.5 mg/dL, normal range: 10–40 mg/dL). Anti‐Ro52 antibody (49 RU/mL) and anti‐ribosomal P (Rib‐P) proteins antibody (80 RU/mL) were positive whereas anti‐Sjögren's Syndrome‐related antigen A (SS‐A) antibody (4 RU/mL), anti‐Sjögren's Syndrome‐related antigen B (SS‐B) antibody (2 RU/mL), anti‐Jo 1 antibody (0 RU/mL), anti‐Scl 70 antibody (0 RU/mL), anti‐centromere protein B (CENP‐B) antibody (1 RU/mL), and rheumatoid factor were negative. Due to his poor cooperation, lung function tests could not be completed. Echocardiography revealed mild concentric left ventricular hypertrophy, dilated left ventricle, and normal left ventricular systolic and diastolic function with an ejection fraction of 65%.

**FIGURE 2:**
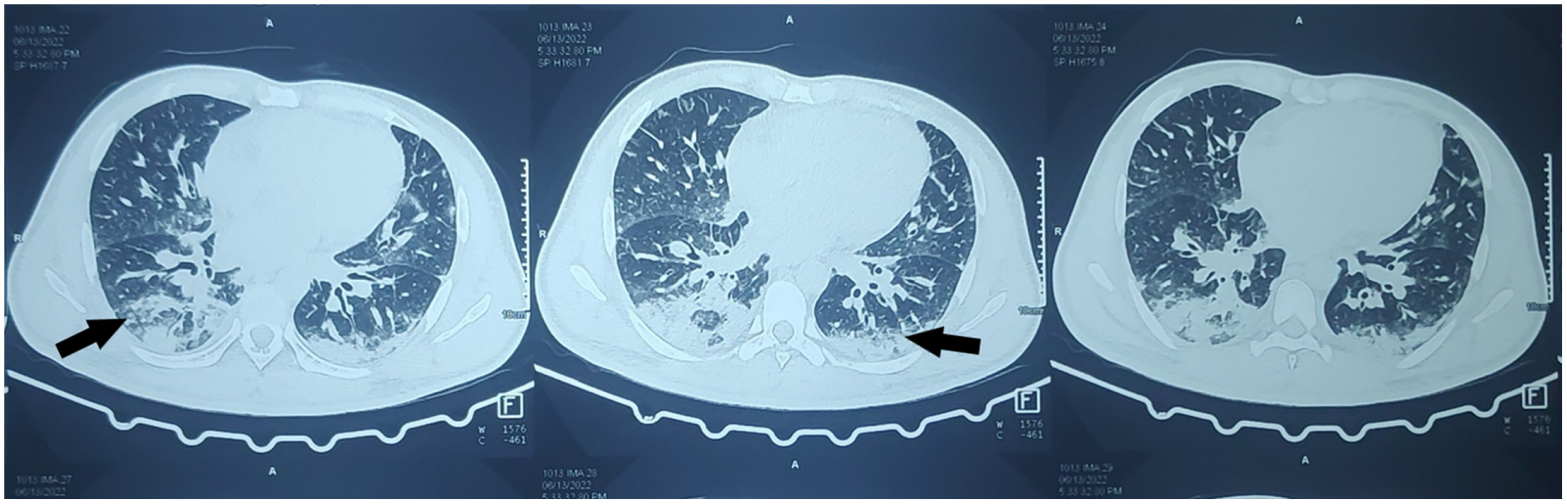
Axial image of high‐resolution computed tomography (HRCT) of chest shows areas of patchy and confluent areas of dense consolidation with surrounding ground glass opacities involving bilateral lower lobes (arrows).

High‐resolution computed tomography (HRCT) of the chest showed patchy and confluent dense peripheral consolidation with surrounding ground‐glass opacities involving bilateral lower lobe suggesting organizing pneumonia as shown in Figure 2.

The differentials in our case included infections, malignancy, and autoimmune diseases like SLE, rheumatoid arthritis, overlap syndromes, mixed connective tissue disease, and vasculitis. Based upon the clinical, radiological, and immunological findings and after ruling out other causes, diagnosis of SLE‐related OP was made.

He was treated with intravenous methylprednisolone of 1 g daily for 3 days followed by 1 mg/kg daily and an injection of cyclophosphamide 750 mg monthly. He is currently asymptomatic with a marked resolution of lung opacities in follow‐up imaging.

## 3 DISCUSSION

SLE, primarily occurring in young women, is a chronic autoimmune condition and one of the connective tissue diseases (CTD) affecting the diverse organ systems and is related to an encyclopedic range of manifestations.^4^ Frequent pulmonary exemplifications of SLE include pleuritis (40%–60%), pleural effusion (50%), acute pneumonitis (1%–12%), interstitial lung disease (3%–9%), shrinking lung syndrome (1%–6%), pulmonary hypertension (4%), diffuse alveolar hemorrhage, and thromboembolic disease.^5^ Only 3% of the patients were reported to have lung involvement at the onset of lupus while 7% of patients developed pulmonary presentation during the course of observation, in a prospective study by Cervera et al.^5^, ^6^ ILDs are a well‐described complication of CTD and encompass a heterogeneous group of disorders with various etiologies.^7^ Histopathological patterns include non‐specific interstitial pneumonia (NSIP), usual interstitial pneumonia (UIP), and desquamative interstitial pneumonia (DIP).^7^, ^8^, ^9^ NSIP is the most common form of ILD seen in SLE.^8^

OP is considered to be an inflammatory process rather than a fibrosing disease and is characterized by the establishment of plugs of fibrous tissue within alveoli, alveolar ducts, and distal bronchiole typically accompanied by inflammatory changes involving bronchioles and surrounding interstitium.^10^, ^11^ Patient's exposure to various drugs and infective etiology have to be excluded before attributing OP secondary to CTD.^10^ Almost half of the patients with OP present with persistent non‐productive cough, shortness of breath, low‐grade fever, malaise, and weight loss, and infrequent symptoms include chest pain and hemoptysis.^11^ Pulmonary function tests may show a restrictive pattern.^11^ Physical examination may reveal fine crackles.^11^ In our case, the patient presented with fever, dry cough, weight loss, myalgia, and chest pain, and had fine crackles on auscultation.

The preferred diagnostic method in OP is lung biopsy but in the current era the diagnosis is usually guided by clinical and radiological findings.^10^, ^12^ As lung biopsy is performed in less than 15% of the patients, provided the infective etiology is ruled out, conventional radiography and computed tomography (CT) can serve as initial diagnostic modalities for OP.^12^, ^13^ Bilateral patchy infiltrates, small linear opacities or reticulonodular opacities can be seen in chest imaging in OP.^14^ Tadaka et al. noticed that OP might be diagnosed early with a greater frequency with using lung CT thereby proceeding in early institution of treatment.^3^ Further, a multicenter study of SLE‐associated interstitial pneumonia patients showed that 22% (14/55) had OP on a HRCT chest.^13^ The absence of honeycombing or an irregular reticular pattern can assist in differentiating OP from other ILDs in CT.^12^, ^14^ It was reported that ILD with SLE is hugely related to the presence of anti‐SS‐A (Ro) antibodies, and 82% of patients (9/11) with lupus pneumonitis had anti‐SS‐A (Ro) antibodies.^15^ Our patient was also positive for anti‐Ro antibodies in the immunological testing. The exact etiology of OP in SLE remains ambiguous.^16^, ^17^ The presence of immunoglobulin G (IgG) and immunoglobulin M (IgM) deposits lining the alveolar walls, infiltration of plasma cells in the bronchiolar walls, and favorable reaction to steroid treatment point toward a possible immunological mechanism resulting in epithelial harm and consequently leading to the development of OP.^16^, ^17^

Even though there is widespread use of corticosteroids in treatment of OP, its efficacy still remains unclear.^18^ Tadaka et al. described significant improvement after treatment with corticosteroid in one patient.^3^ Variable response to corticosteroids was found in a case series as one patient had a good response and others had fatal outcomes even after using steroids.^19^ Eleven SLE patients diagnosed with OP after lung biopsy had significant clinical improvement with corticosteroids.^20^ Our patient was treated with corticosteroid and cyclophosphamide which resulted in significant improvement. Further studies are needed to delineate the pathophysiology, clinical course, and prognosis of OP in SLE.

## 4 CONCLUSION

Although rare, organizing pneumonia as an initial presentation in lupus can also be diagnosed with the help of CT even before the report of infrequently performed lung biopsy is available. This can lead to early initiation of immunosuppressive therapies which help to reduce potential complications and disease evolution thereby improving prognosis.

## AUTHOR CONTRIBUTIONS

**Suman Acharya:** Conceptualization; data curation; writing – original draft; writing – review and editing. **Roshan Aryal:** Data curation; writing – original draft; writing – review and editing. **Rupesh Kumar Yadav:** Writing – original draft; writing – review and editing. **Suraj Shrestha:** Writing – original draft; writing – review and editing. **Rikesh Karki:** Writing – original draft; writing – review and editing. **Saket Jha:** Conceptualization; supervision.

## FUNDING INFORMATION

Not available.

## CONFLICT OF INTEREST STATEMENT

The authors have no conflict of interest declare.

## ETHICS APPROVAL AND CONSENT TO PARTICIPATE

Not required.

## CONSENT FOR PUBLICATION

Written informed consent was obtained from the patient for publication of this case report and accompanying images. A copy of the written consent is available for review by the Editor‐in‐Chief of this journal on request.

## Data Availability

All the necessary data and materials are within the manuscript.
